# Acute flaccid myelitis and enterovirus D68: lessons from the past and present

**DOI:** 10.1007/s00431-019-03435-3

**Published:** 2019-07-23

**Authors:** Jelte Helfferich, Marjolein Knoester, Coretta C. Van Leer-Buter, Rinze F. Neuteboom, Linda C. Meiners, Hubert G. Niesters, Oebele F. Brouwer

**Affiliations:** 1 Department of Neurology, University Medical Center Groningen, University of Groningen, Hanzeplein 1, PO Box 30001, 9700 RB Groningen, The Netherlands; 2 Department of Medical Microbiology and Infection Prevention, Division of Clinical Virology, University Medical Center Groningen, University of Groningen, Groningen, The Netherlands; 3 Department of Pediatric Neurology, Erasmus Medical Center, Erasmus University, Rotterdam, The Netherlands; 4 Department of Radiology, University Medical Center Groningen, University of Groningen, Groningen, The Netherlands

**Keywords:** Acute flaccid myelitis, Acute flaccid paralysis, Enterovirus D68, Poliovirus, Poliomyelitis, Enterovirus

## Abstract

Acute flaccid myelitis is characterized by the combination of acute flaccid paralysis and a spinal cord lesion largely restricted to the gray matter on magnetic resonance imaging. The term acute flaccid myelitis was introduced in 2014 after the upsurge of pediatric cases in the USA with enterovirus D68 infection. Since then, an increasing number of cases have been reported worldwide. Whereas the terminology is new, the clinical syndrome has been recognized in the past in association with several other neurotropic viruses such as poliovirus.

*Conclusion*: This review presents the current knowledge on acute flaccid myelitis with respect to the clinical presentation and its differential diagnosis with Guillain-Barré syndrome and acute transverse myelitis. We also discuss the association with enterovirus D68 and the presumed pathophysiological mechanism of this infection causing anterior horn cell damage. Sharing clinical knowledge and insights from basic research is needed to make progress in diagnosis, treatment, and prevention of this new polio-like disease.

**Table:** 

**What is Known:** • *Acute flaccid myelitis (AFM) is a polio-like condition characterized by rapid progressive asymmetric weakness, together with specific findings on MRI* *• AFM has been related to different viral agents, but recent outbreaks are predominantly associated with enterovirus D68.* **What is New:** • *Improving knowledge on AFM must increase early recognition and adequate diagnostic procedures by clinicians.* *• The increasing incidence of AFM urges cooperation between pediatricians, neurologists, and microbiologists for the development of treatment and preventive options.*

**Electronic supplementary material:**

The online version of this article (10.1007/s00431-019-03435-3) contains supplementary material, which is available to authorized users.

## Introduction

Acute flaccid myelitis (AFM) is a syndrome characterized by acute flaccid paralysis (AFP) and gray matter spinal cord lesions on magnetic resonance imaging (MRI). After the introduction of the term AFM in 2014, more than 500 patients, predominantly children, have been recognized both in- and outside Europe [5, 27, 32, 48].

The Center for Disease Control and Prevention (CDC) proposed a case definition in which a definite AFM case is described as acute-onset flaccid weakness, combined with a spinal cord lesion on MRI, largely restricted to the gray matter and spanning one or more spinal segments. Acute flaccid weakness combined with cerebrospinal fluid (CSF) pleocytosis without lesions on MRI is defined as a probable case [4].

A prodromal illness, asymmetric limb weakness, and specific findings in electromyography and nerve conduction studies may further aid in distinguishing AFM from other causes of AFP such as Guillain-Barré syndrome (GBS) and acute transverse myelitis (ATM) [10].

Accumulating evidence supports an association between enterovirus D68 (EV-D68) and AFM [9, 34]. Other viruses that have been associated with outbreaks of acute flaccid weakness and myelitis include enterovirus A71 (EV-A71), West Nile virus (WNV), Japanese encephalitis virus, and the wild-type poliovirus [12, 22, 43, 45].

In this review, we describe the clinical syndrome of AFM, its differential diagnosis, and its association with different viruses, with the emphasis on EV-D68.

## Methods

For this review, we performed a literature search in PubMed on “flaccid myelitis” and “Enterovirus D68” from 2000 until February 2019. A total of 995 titles of articles in English were screened and selected based on relevance for epidemiology, clinical characteristics, pathophysiology, treatment, prevention, and prognosis of AFM. Only cohorts containing at least five children were selected (Table 1).

**Table 1:** Summary of cohorts of children of AFM described after 2012, showing patient characteristics, clinical findings, and findings on further investigations

	Author	Inclusion period	Country/region	No pts	EV-D68 pos	Gender (% male)	Age (mean or median with range)	Prodrome (%)	Limb weakness (%)	Asymmetry (%)	Sensory involvement (%)	Hyporeflexia (%)	Cranial nerve dysfunction (%)	Ventilatory support (%)	Bowel/bladder dysfunction (%)	CSF pleocytosis (%)	Protein raised in CSF (%)	MRI spine: T2 hyperintensity (%)	Nerve root enhancement (%)	Brainstem lesions
1	Andersen	2001–2014	Australia	8	0% (13% EV-A71)	25	Med 5	100	100	100	0	NS	25	NS	0	85	71	100	38	25
2	Messacar	2012–2015	USA	159^a^	20–45%	56–91	Med 7.1 (0.4–73)	64–100	83–100	47–70	21–44	80–81	18–83	9–34	18–51	64–91	45–58	90–100	20–40	35–75
3	Elrick	2012–2016	USA	34	13%	65	Med 5 (< 1–15)	100	100	97	0	67	> 24	24	6	97	45	100	38	62
4	Yea	2014	Canada	25	28%	64	Med 7.8 (0.8-15.0)	88	100	NS	12	88	> 20	28	36	72	28	100	72	32
5	Gordon-Lipkin	2014–2017	USA	16	23%	69	Med 4 (3–6)	100	100	NS	6	63	50	31	NS	100	NS (Med 6.3 g/L)	100	13	42
6	Chong	2015	Japan	59	15%	59	Med 4.4 (2.6–77)	97	100	68	20	90	17	8	27	85	46	100	51	42
7	Knoester	2015–2016	Europe	29	100%^b^	52	Med 4 (1.6–55)	92	100	NS (usual)	7	87	60	66	7	91	NS (Med 3.8 g/L)	92	16	68
8	Bonwitt	2016	USA	10	20% (10% EV-A71)	70	Med 6 (3–14)	80	100	NS	NS	NS	30	10	50	78	NS (Med 5.8 g/L)	100	0	30
9	Iverson	2016	USA	5	60%	20	Mean 7.7 (3.5–12)	100	100	NS	NS	NS	80	NS	NS	100	NS	80	NS	NS
10	Hübner	2016	Germany	16 (7)^c^	6%	50	Mean 4.6 (1.7-14.3)	100	100	86	NS	NS	NS	14	NS	43	NS	86	NS	NS
11	Ruggieri	2016	Argentina	11	36%	54	Mean 3.2 (0.3–6)	100	100	81	0	100	45	36	0	63	18	100	NS	45
12	Sarmast	2017	India	9	0%	56	Med 5.5 (2–7)	100	100	100	0	100	11	NS	0	89	22	100	NS	11
13	McKay	2018	USA	80	37% (29% EV-A71)	59	Med 4 (0.7–32)	99	100	NS	NS	NS	NS	NS	NS	83	NS (Med 4.7 g/L)	100	NS	NS
14	Ramsay	2018	UK	40 (16)^d^	36%	53	55% under 5 yo	55	98	NS	NS	NS	NS	55	NS	18	NS	43	NS	NS

*No pts*, number of patients; *EV-D88*, enterovirus D68; *EV-A71*, enterovirus A71; *med*, median; *CSF*, cerebrospinal fluid; *MRI*, magnetic resonance imaging; *USA*, United States of America; *UK*, United Kingdom; *NS*, not specified; *yo*, year old

^a^Combination of four US cohorts with a partial overlap in these cohorts

^b^EV-D68 had to be identified for inclusion

^c^16 registered cases, 7 of which were further described

^d^40 cases of Acute Flaccid Paralysis, of which 16 fulfilled the criteria for probable or definite AFM

### Epidemiology

Before the term AFM was introduced, outbreaks of acute flaccid weakness and myelitis, matching the case definition for AFM, were reported in association with EV-A71, predominantly in Eastern Asia and Australia, and with WNV, causing several outbreaks in the USA in the beginning of this century [12, 22]. Poliomyelitis also matches the case definition of AFM and can be seen as the first known cause of AFM. However, MRI was and is often not available in countries where poliomyelitis still occurs, making the definite diagnosis of AFM difficult.

In 2012, the first probable cases were reported in California (USA) [10]. Since 2014, the CDC has reported over 500 cases of AFM in the USA with 2-year intervals and several cohorts of patients with AFM have been reported worldwide (Table 1) [5, 10, 11, 23, 24, 31, 39, 40, 42, 48]. A recent study reported an incidence of 1.46 per 100,000 person years, although reliable data is lacking, as AFM is notifiable in only few countries and the clinical picture is often not recognized [25].

In different cohorts of AFM patients, EV-D68 was detected in 20–40% of cases, primarily from respiratory specimens (Table 1). The variation in detection percentages might be explained by differences in timing and performance of diagnostic procedures and by selection criteria for patients [25]. Most reported patients with AFM were children under the age of 10 with a slight male preponderance. A majority were previously healthy, but asthma was seen in 12–32% of children [25, 33, 48].

Both EV-A71 and West Nile virus are still circulating and have also been detected in recent cohorts of AFM patients [2, 3, 31]. Outbreaks of poliomyelitis are currently rare, due to a global poliovirus surveillance and vaccination program [8].

### Clinical features

The clinical characteristics of non-polio AFM cohorts described in literature since 2012 are summarized in Table 1. Muscle weakness typically develops over the course of several hours to days, often with a marked asymmetry. Weakness is proximally usually more severe and may be more pronounced in the upper limbs, with a spectrum of severity varying between slight paresis of a single limb to tetraplegia. Tendon reflexes are typically diminished or absent in the affected limbs. In most patients, there is a prior prodromal illness, often involving the upper respiratory tract, with a median of 5 days before onset of weakness [5, 10, 27, 33].

Weakness can be limited to the extremities, but the diaphragm and bulbar muscles may also be affected, making ventilatory support necessary in the acute phase in about 30% of cases [10, 13, 33, 48]. Cranial nerve deficits are common and may be the only finding. The facial nerve is most often affected, followed by the abducens and oculomotor nerves [33].

Associated features include severe limb pain and autonomic disturbances such as bladder dysfunction. Sensory symptoms, primarily paresthesia, are reported in up to 20% of cases [5, 10, 27, 33].

The clinical features of cohorts of AFM, described before 2012, associated with EV-A71, WNV, and poliovirus were highly similar, although poliovirus-related AFM more often affected lower limbs, with bulbar muscles usually being spared [43]. EV-A71 has also been associated with rhombencephalitis, sometimes with severe cardiorespiratory symptoms [12, 22, 43].

### Differential diagnosis

AFM is included in the broad differential diagnosis of AFP. AFP is defined as a syndrome of focal weakness of peripheral origin in any part of the body with an acute onset [30].

It is important to be able to recognize AFM early in its course so that adequate diagnostic procedures can be performed and respiratory failure in the initial phase can be anticipated. Both clinical clues and findings on further investigations may help differentiate AFM from other causes of AFP.

In cases of AFM in which only one arm is affected, the initial thought may be that of synovitis or arm injury. Clinical clues that may help in distinguishing these from AFM may be the presence of a prodromal illness, the hypo- or areflexia, and the often-associated neck weakness in AFM.

When more than one limb is affected, the differential diagnosis includes other causes of acute myelopathy, such as acute transverse myelitis (ATM), acute disseminated encephalomyelitis (ADEM), acute cord compression, and ischemic myelopathy. Furthermore, Guillain-Barré syndrome (GBS) may be suspected because of the sudden onset of flaccid weakness after a prodromal illness.

**Fig. 1:**
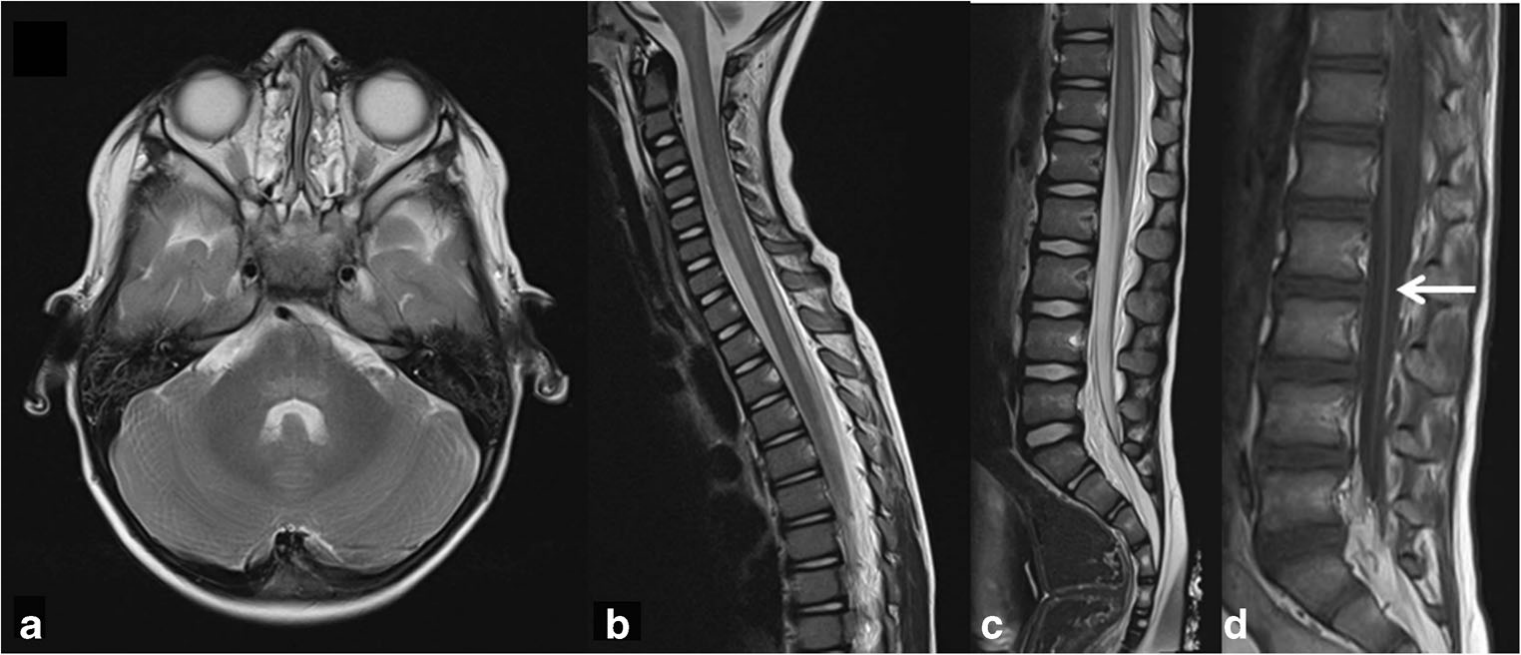
MRI of the neuraxis in a 3-year-old boy with EV-D68–associated AFM. **a** Brain: transverse T2-weighted image showing an area of slight hyperintensity in the dorsal pons (arrow). **b** and **c** Spinal cord: sagittal T2-weighted images showing longitudinal slight hyperintensity largely restricted to the central cord, where the gray matter is situated (arrow). **d** Spinal cord: contrast enhancement of the ventral caudal roots on a sagittal T1-weigthed image (arrow) (republished with permission from [16])

**Fig. 2:**
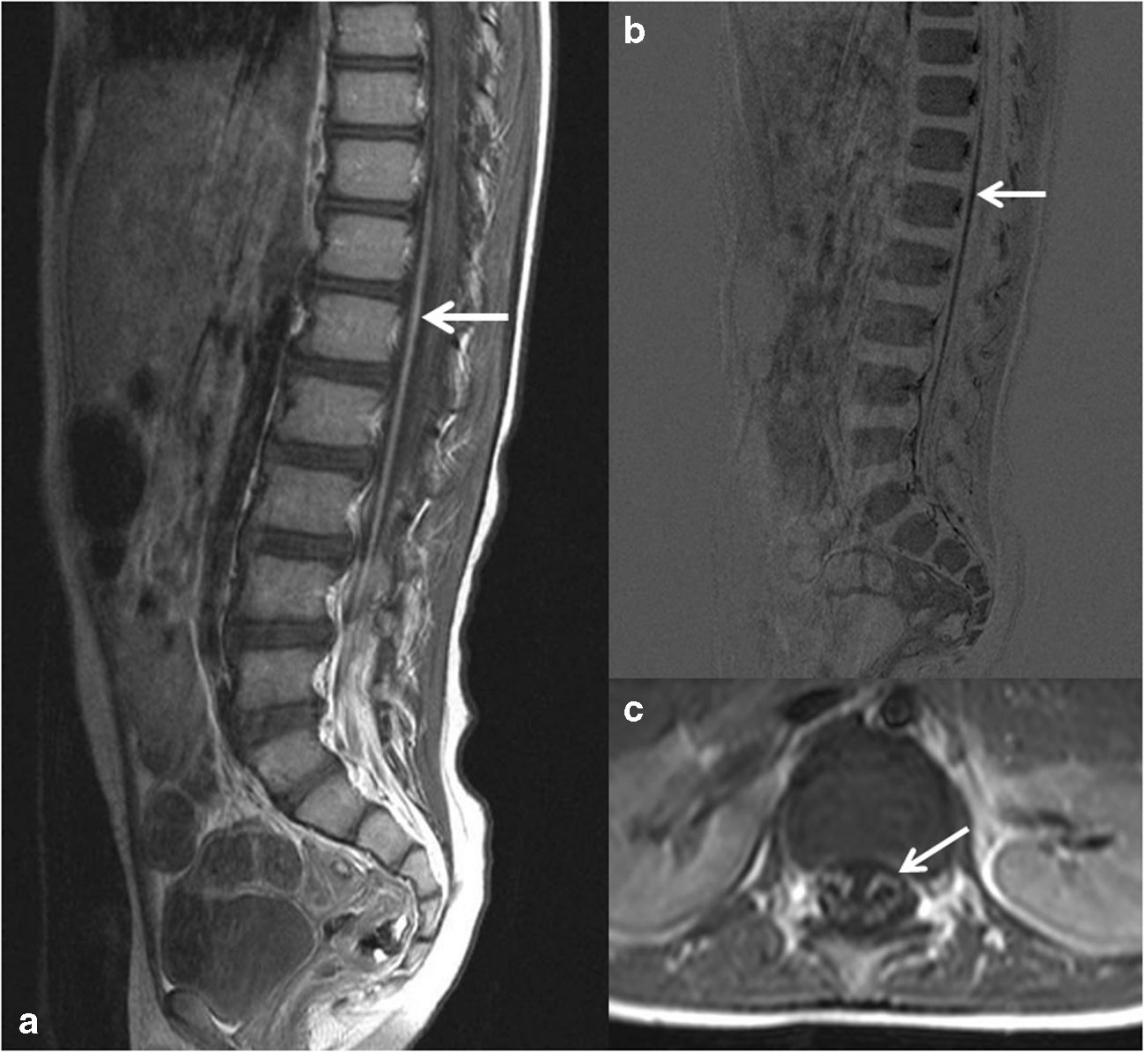
MRI of the spinal cord in a 3-year-old boy with Guillain-Barré syndrome. **a** Sagittal contrast-enhanced T1 showing typical enhancing anterior caudal roots. **b** Subtraction of A with more clear depiction of enhancing caudal root. **c** Transverse T1 showing more clear enhancement of anterior motor roots

**Fig. 3:**
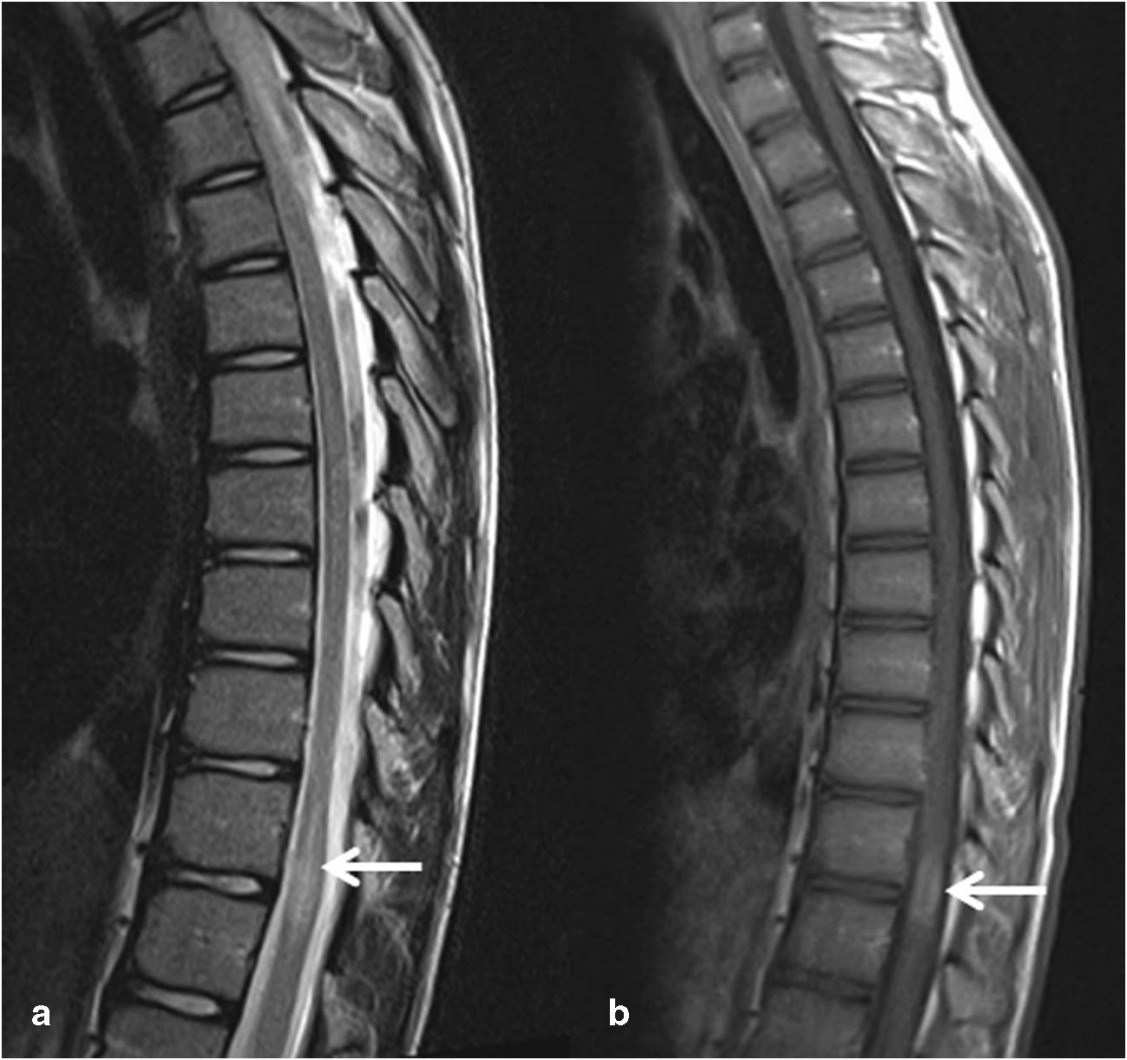
MRI of the spinal cord in a 15-year-old boy with acute transverse myelitis, eventually diagnosed with relapsing remitting multiple sclerosis. **a** Sagittal T2 showing focal swelling of the spinal cord at level Th11–12. **b** Sagittal T1 showing contrast enhancement of the lesion

**Fig. 4:**
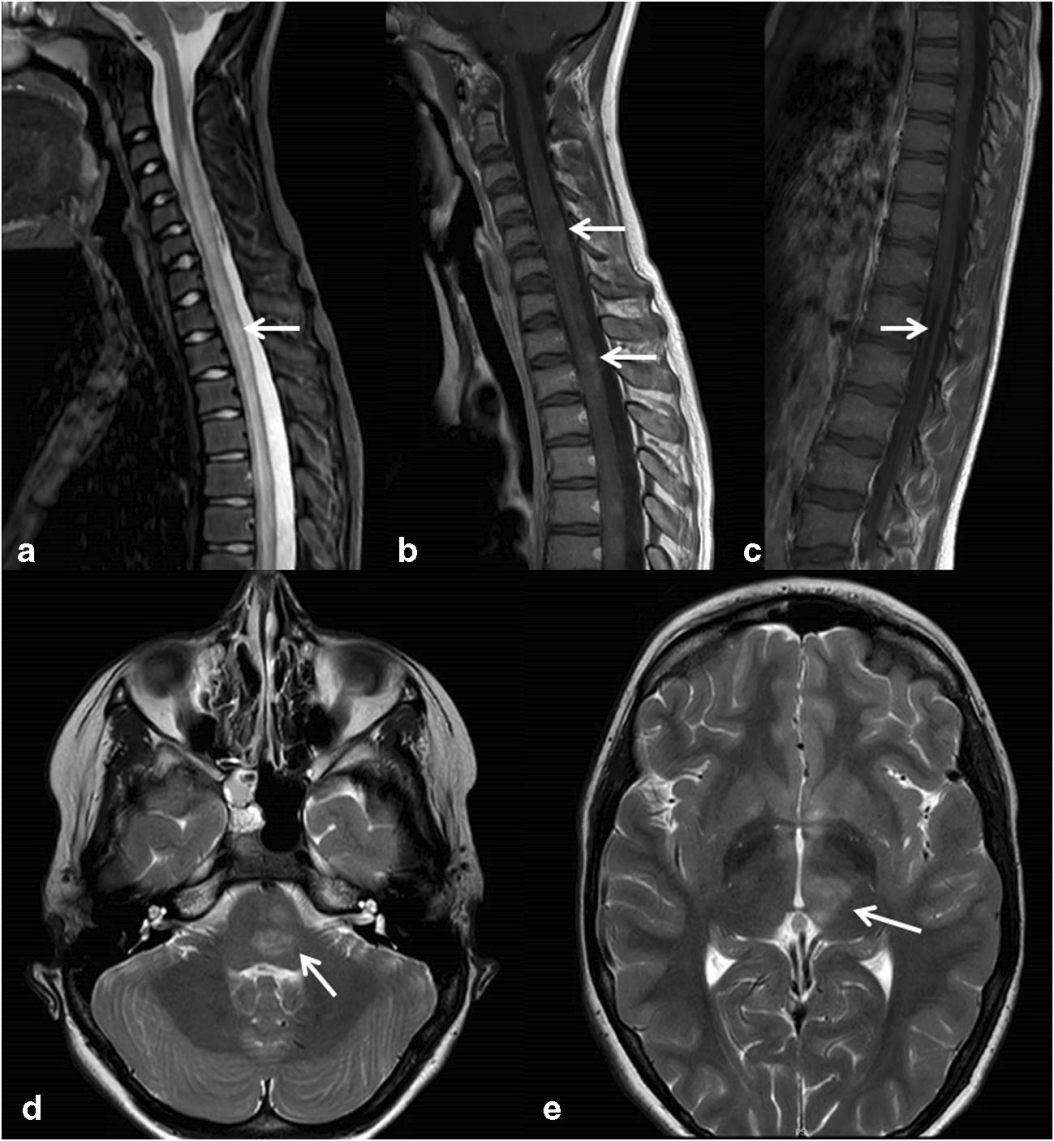
MRI of a 13-year-old boy with a provisional diagnosis of acute demyelinating encephalomyelitis. **a** Sagittal short tau inversion recovery (STIR) with edematous cervicothoracic spinal cord from the level of C4. **b** Sagittal T1 of the spinal cord showing diffuse areas of slight enhancement. **c** Enhancement of mainly dorsal roots in a sagittal T1 of the lumbar spine. **d** and **e** Transverse T2 at the level of the pons **(d)** and thalamus **(e)** showing asymmetric hyperintense areas.

While the asymmetric weakness, the absence of encephalopathy, the paucity of sensory symptoms, and the presence of cranial nerve deficits in AFM may help in distinguishing it from other causes of AFP, further investigations are required to make the right diagnosis (Table 2; Figs. 1, 2, 3, 4) [1, 10, 20, 33, 47].

**Table 2:** Signs, symptoms and findings on further investigations in acute flaccid myelitis, Guillain-Barré syndrome, and acute transverse myelitis

	Acute flaccid myelitis (with EV-D68)	Guillain-Barré syndrome	Acute transverse myelitis
Prodrome			
Type	Febrile illness often with respiratory and/or gastrointestinal symptoms	Febrile illness often with gastrointestinal symptoms and or respiratory symptoms	Commonly a preceding febrile illness
Time until onset of weakness	Usually within 1 week	Several weeks	Days to weeks
Clinical details			
Neurologic deficits	Asymmetric flaccid weakness, with upper limbs often more affected, proximal > distal	Ascending weakness, lower limbs > upper limbs	Symmetric weakness, may be asymmetric initially
Reflexes	Typically low or absent	Low or absent	Usually high, can be low initially
Sensory symptoms	Typically no sensory deficits	Paresthesia and slight distal sensory symptoms (except in AMAN)	Common, often with a sensory level
Cranial nerve deficits	Bulbar weakness and asymmetric facial palsy common; sometimes oculomotor deficits	Symmetric facial weakness; oculomotor deficits in MFS	None
Other symptoms	Pain, autonomic dysfunction	Pain, autonomic dysfunction	Bowel and bladder dysfunction
Time course	Progressive over hours to days	Progressive symptoms over several days	Progressive over 4 h to 21 days
Findings			
CSF	Slight pleocytosis, raised protein. May be completely normal	Raised protein after several days, without pleocytosis (“dissociation cytoalbuminique”)	Slight pleocytosis, raised protein. May be completely normal
Microbiology	EV-D68 in respiratory specimen	*Campylobacter jejuni* in feces; EBV, CMV, HEV, Zika virus in blood	Usually none
MRI brain	Typical T2-hyperintense region in the dorsal pons, sometimes also in caudate nuclei. Cranial nerve enhancement possible	Normal	Normal
MRI spine	Longitudinally extensive diffuse slightly hyperintense central cord lesion, usually most pronounced in the cervical region. Sometimes cauda equina root enhancement	Cauda equina root enhancement may be found	Central cord focal hyperintense lesion over multiple levels affecting white and gray matter
EMG	Findings of motor axonopathy with low CMAPs, normal NCV. Normal sensory findings	Decreased NCV with blocks are typical. Normal sensory findings in AMAN	Normal
Treatment/prognosis			
Treatment	No effective treatment, potential positive effect of IVIG	IVIG and/or plasmapheresis effective	High-dose steroids, sometimes IVIG and/or plasmapheresis
Prognosis	Improvement over several months, but often significant residual weakness and muscle atrophy	Often complete recovery over the course of weeks until months	Partial recovery over the course of months until years

*EV-D68*, enterovirus D68; *AMAN*, acute motor axonal neuropathy; *MFS*, Miller Fisher syndrome; *EBV*, Epstein Barr virus; *CMV*, cytomegalovirus; *HEV*, hepatitis E virus; *CMAP*, compound muscle action potential; *NCV*, nerve conduction velocity; *IVIG*, intravenous immunoglobulin

### Investigations

Diagnostic tests recommended in children with suspected AFM should be directed at the identification of different microorganisms and the exclusion of other causes (Table 3) [15]. Initial investigations must be performed on blood, stool, respiratory material, and CSF, followed by MRI of the brain and spinal cord and in some cases electromyography (EMG).

**Table 3:** Suggested workup for children with acute flaccid paralysis

Blood	Routine investigations (blood count, inflammatory parameters, creatine kinase, liver and renal function tests)
	Auto-antibodies (anti-MOG IgG, anti-AQP4, anti-GM1, anti-GQ1b)
	Oligoclonal bands (both serum and CSF)
	Microbiology: testing for enterovirus (including poliovirus), EBV, CMV, VZV, HEV, Zika virus*
CSF	Routine investigations (cell count, protein, glucose)
	Oligoclonal bands (both CSF and serum)
	Microbiology: testing for enterovirus, parechovirus, HSV, VZV, EBV
Further microbiologic testing	Nasopharyngeal swab for enterovirus testing Stool sample for enterovirus and *C. jejuni* testing
Imaging	Contrast-enhanced MRI of the brain and spine
Neurophysiologic testing	EMG with motor and sensory investigation of an affected limb

*MOG*, myelin-oligodendrocyte glycoprotein; *AQP4*, aquaporin 4; *GM1*, ganglioside M1; *GQ1b*, ganglioside Q1b; *EBV*, Ebstain Barr virus; *CMV*, cytomegalovirus; *VZV*, Varicella Zoster virus; *HEV*, hepatitis E virus; *CSF*, cerebrospinal fluid; *HSV*, Herpex Simplex virus; *EMG*, electromyography

*For patients that have traveled to or live in countries where Zika virus is prevalent

#### Blood

General laboratory investigation of blood samples of AFM patients may show a slight leukocytosis, sometimes with raised inflammatory parameters, which is usually not helpful in the differentiation of AFM from other disorders causing AFP [20, 33].

#### Cerebrospinal fluid

CSF examination in AFM patients in the described cohorts since 2012 reveals a mild to moderate pleocytosis in most cases (Table 1). Protein levels are initially minimally raised in about half of AFM cases but can be completely normal. After several days, the leukocyte number tends to decrease, while protein levels rise [5, 14, 27, 33]. Oligoclonal bands in the CSF can be identified in immune-mediated conditions such as ATM, but are usually not found in AFM [1].

Interestingly, viral agents, such as EV-D68, EV-A71, and poliovirus, are only detected in the CSF in a small minority of patients with AFM [5, 22, 27, 40, 43].

#### Virology diagnostic testing

The viral RNA of EV-D68 is detected mostly in respiratory samples, followed to a much lesser extent by feces and can only rarely be found in blood or CSF. This in contrast to EV-A71, which is more frequently detected in blood, and poliovirus, which is routinely identified in stool samples [15, 43].

Obtaining an adequate respiratory sample is therefore indispensable for detection of EV-D68. Considering the fact that the prodromal, mostly respiratory illness is usually a few days into its natural course when a patient presents with weakness, the best chances of detecting EV-D68 is soon after onset of complaints. Several PCR tests have been described, which test either directly for EV-D68 or for enteroviruses in general [15, 38].

#### Magnetic resonance imaging

MRI of the brain and spinal cord is important in making the diagnosis of AFM and in distinguishing it from other causes of AFP (Table 2 and Figs. 1, 2, 3, 4) [13]. CT usually shows no abnormalities [28].

In AFM, the classical MRI feature is a longitudinally extensive slight T2-hyperintense signal in the central cord, affecting the central gray matter, often most pronounced in the cervical regions (Fig. 1b, c). Initially, there is usually more diffuse spinal cord edema, evolving over several days to T2-hyperintensity that is restricted to the anterior horn. Enhancement of the caudal roots and sometimes of the cranial nerves can be seen (Fig. 1d) [28].

MRI of the brain commonly reveals an area of slight hyperintensity typically located in the dorsal pons in the region of the nuclei of the abducens and facial nerve. The corticospinal tracts, located ventrally, are not affected (Fig. 1a), while the caudate nucleus may be involved [28]. These findings may help in securing the diagnosis, but the correlation between symptoms and radiologic findings is usually poor, making MRI unsuitable as a prognostic tool for AFM [37].

Imaging findings in earlier outbreaks of AFM, associated with WNV and poliovirus, were highly similar, while in EV-A71–associated neurological disease, these appear to be more variable and more extensive brain abnormalities may occur [6, 12, 22].

#### Neurophysiological studies

While EMG findings in recent outbreaks of AFM can be normal on the first day, after several days, a pattern compatible with anterior horn disease is seen. This encompasses decreased compound muscle action potentials (CMAP) with normal conduction velocities. Sensory testing is usually completely normal [21].

After some weeks, denervation potentials can be seen, with severe ongoing denervation being a possible predictor for the gravity of residual damage. The persistence of F-waves may indicate a better prognosis [5, 21, 33].

### Virology

Enteroviruses, such as EV-D68, EV-A71, and poliovirus, are small RNA viruses belonging to the picornavirus family. EV-D68 was first identified in 1962 after isolation from children with severe respiratory disease [44]. Since 2012, an increasing incidence has been recognized, with infections mostly occurring in autumn and late summer. EV-D68 appears to occur in a cyclic pattern with a 2-year interval [27, 34].

EV-D68 infection may be asymptomatic or cause respiratory disease. In hospitalized children, an asthma-like respiratory disease is most commonly seen [39]. The percentage of infected patients afflicted with paralytic disease is not yet known, but is estimated to be less than 1%, similar to poliomyelitis [29, 43].

### Pathophysiology

A causal relationship between EV-D68 and AFM is supported by epidemiological and biological evidence, as was evaluated by different groups applying the Bradford Hill criteria [9, 34].

The biological evidence mainly came from mouse models, in which mice infected with contemporary circulating strains of EV-D68 develop flaccid paralysis mimicking AFM. Interestingly, neonatal or young mice are used, because older mice are not susceptible to disease [17, 36]. Pathologic examination of infected mice revealed the presence of the virus in the anterior horn with associated cell loss [17, 36]. EV-D68 probably reaches the anterior horn by retrograde axonal transport, as is supported by both mouse studies and in vitro studies in human motor neurons [17, 19, 36].

One study found myositis without spinal cord infection after intranasal injection of the virus in mice [36].

Although the results from mouse studies cannot simply be extrapolated to humans, these results are suggestive of a damaging effect of the virus in anterior horn cells, possibly combined with a direct damaging effect on muscles through viral myositis.

Important questions remain why only some EV-D68 infected patients develop AFM and how the variability in severity of AFM in affected patients is explained.

### Treatment

There are currently no effective treatment options for AFM. Most patients are treated with intravenous immunoglobulin (IVIG), steroids, or plasmapheresis, or a combination, but no significant clinical effect of any of these interventions has been shown so far. Because of its effectiveness in the mouse model of EV-D68–associated AFM and its possible efficacy in treatment of EV-A71–associated encephalomyelitis, treatment with IVIG has been recommended [18, 33, 46, 48].

The anti-inflammatory effects of steroids may be beneficial in AFM cases with spinal cord edema or white matter involvement, but steroids are unlikely to be effective in limiting the anterior horn damage that is probably caused by a direct damaging effect of the virus. Furthermore, treatment with steroids in a mouse model of AFM associated with EV-D68 led to an increased viral load and a deterioration of motor symptoms [17, 36].

Fluoxetine, an antidepressant, is effective in inhibiting EV-D68 replication in vitro. However, treatment with fluoxetine in the mouse model of EV-D68–associated AFM did not result in reduction of the viral load or improvement of motor function. Also, no significant effect has been shown in patients with AFM, treated with fluoxetine [18, 35].

While scientific proof is still lacking, we recommend IVIG in the acute phase, combined with maximal supportive care with optimal pain control, feeding, ventilatory support, and intensive rehabilitation. Surgical procedures such as nerve and muscle transfers have been performed and cases have been described in which improvement of limb function has been achieved. Because over time degeneration of the receiving motor nerves and muscle fibers will occur, evaluation for surgical intervention should be considered early in the disease course [41].

### Prevention/vaccination

In the mouse model of EV-D68–associated AFM, passive immunization with pooled immune sera, if administered before injection of the virus, was effective in decreasing the rate of paralysis [18].

Arguments for vaccination as a treatment strategy arise from the development of effective vaccines against EV-A71 infections in China and the effective eradication of poliomyelitis in most of the world after introduction of vaccination [8, 49]. Recently, an experimental vaccine based on virus-like particles targeting EV-D68 has been developed. This vaccine has been proven effective in a mouse model in the prevention of AFM [7].

### Prognosis

Only 5–39% of patients with AFM recover partially to completely (supplementary table 1). Most patients retain significant residual motor deficits, and prolonged need for ventilatory support is not uncommon. On follow-up, residual proximal weakness tends to be more severe than distal weakness, with severe atrophy occurring over time [5, 13, 23, 26, 29, 48]. Cranial nerve deficits usually recover well over time. Death is uncommon but has been reported in immunocompromised patients, usually because of respiratory complications [27, 33]. While not much is known about prognostic factors, more severe disability and weakness at nadir and the persistence of denervation seem to be associated with worse outcome. One study found a correlation between negative tests for EV-D68 at onset and better outcome, which made the authors speculate that viral clearance and host responses play a role in the severity of weakness in AFM [5]. Alternatively, these EV-D68–negative cases may be due to different etiologies associated with more favorable outcomes than cases confirmed to be associated with EV-D68.

## Conclusion and future perspectives

AFM is a newly introduced term comprising AFP combined with longitudinally extensive lesions of the spinal cord on MRI. This syndrome resembles poliomyelitis and has been associated with different viruses, in particular EV-D68.

EV-D68 infection is usually asymptomatic or mildly symptomatic with respiratory illness, but it can be associated with anterior horn disease causing severe weakness, with only minimal improvement over time in most cases.

A major challenge lies in the propagation of correct diagnostic procedures, including viral testing on respiratory material in suspected AFM cases. Future research may identify risk factors for AFM in EV-D68–infected patients and will elucidate how these factors can be influenced.

We believe that worldwide collaboration between neurologists, radiologists, pediatricians, and microbiologists is necessary to make progress in preventing and treating this devastating childhood disease. Furthermore, we postulate that making AFM a notifiable disease in more countries can increase awareness among clinicians and governments.

## Electronic supplementary material

ESM 1 (DOCX 73 kb)

## Abbreviations

ADEM: Acute disseminated encephalomyelitis
AFM: Acute flaccid myelitis
AFP: Acute flaccid paralysis
ATM: Acute transverse myelitis
CDC: Center for Disease Control and Prevention
CMAP: Compound muscle action potential
CSF: Cerebrospinal fluid
EMG: Electromyography
EV-A71: Enterovirus A71
EV-D68: Enterovirus D68
GBS: Guillain-Barré syndrome
IVIG: Intravenous immunoglobulin
MRI: Magnetic resonance imaging
WNV: West Nile virus
