# Supplementary material for: Acute flaccid myelitis and enterovirus D68: lessons from the past and present
Source: Eur J Pediatr. 2019 Jul 23;178(9):1305–15. doi: 10.1007/s00431-019-03435-3 (PMC6694036; doi:10.1007/s00431-019-03435-3)
Supplement: Supplementary file 1 — (DOCX 73 kb) [file 431_2019_3435_MOESM1_ESM.docx]

| Author | No pts | EV-D68 pos | Follow-up duration (months) | Outcome | Positive prognostic factors | Negative prognostic factors |
| --- | --- | --- | --- | --- | --- | --- |
| Messacar | 159 | 20-45% | Med 4.2-12 | Persistent motor deficits in 75-95% |  |  |
| Chong | 59 | 15% | Med 8,5 | 39% good or complete improvement | Higher pre-treatment muscle strength, normal F-wave persistence, negative EV-D68 identification |  |
| Gordon-Lipkin | 16 | 23% | Med 4 | 38% good recovery (GFMCS I or II), 57% wheelchair bound |  | More severe disability at nadir |
| Martin | 10 | 13% | 12 | 33% full recovery |  | Possibly persistent denervation on follow-up EMG (mean 10.5 months after onset) |
| Yea | 25 | 28% | 3-18 | 8% full recovery, median EDSS 3 |  | Initial EDSS score>4 |
| Knoester | 29 | 100% |  | 11% full recovery, 75% partial recovery |  |  |
| Kirolos | 5 | 100% | 18 | 20% full recovery |  |  |

**Supplementary table**

*Studies showing longer term outcome figures in Acute Flaccid Myelitis*

*EDSS: Expanded Disability Status Scale, EMG: Electromyography, EV-D68: Enterovirus D68, med=median, GFMCS= Gross Motor Function Classification System*
